# Supplementary material for: Fundamental investigations on the ionic transport and thermodynamic properties of non-aqueous potassium-ion electrolytes
Source: Nat Commun. 2023 Jun 28;14:3833. doi: 10.1038/s41467-023-39523-0 (PMC10307903; doi:10.1038/s41467-023-39523-0)
Supplement: Supplementary file 1 — Supplementary Information [file 41467_2023_39523_MOESM1_ESM.pdf]

Supplementary Information

Fundamental Investigations on the Ionic Transport and Thermodynamic Properties of Non-aqueous Potassium-Ion Electrolytes

Shobhan Dhir[1,2], Ben Jagger[1,2], Alen Maguire[1], and Mauro Pasta[1*]

[1]Department of Materials, University of Oxford, OX1 3PH, UK
[2]These authors contributed equally
[*]Corresponding author: mauro.pasta@materials.ox.ac.uk

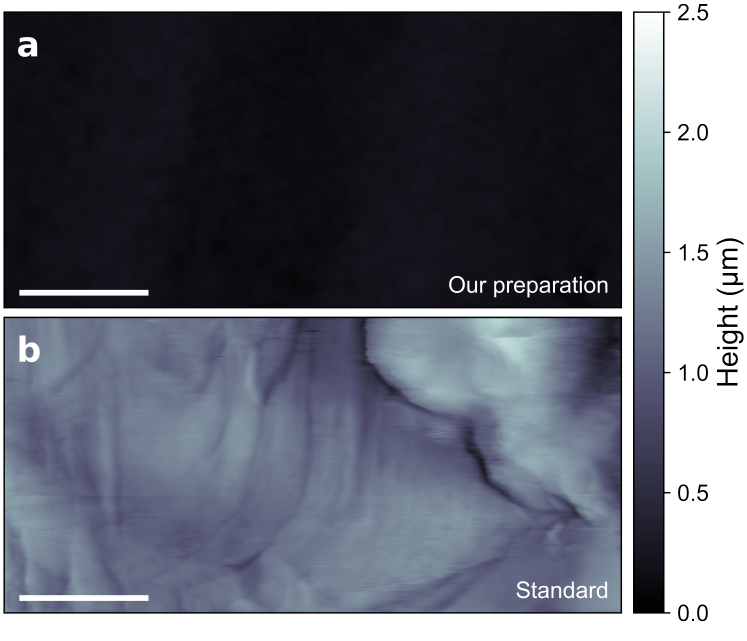

Supplementary Fig. 1 Comparison atomic force microscopy height maps of the surface of pristine K metal samples using (**a**) our preparation protocol (**b**) the standard procedure. Scale bars, 2 μm

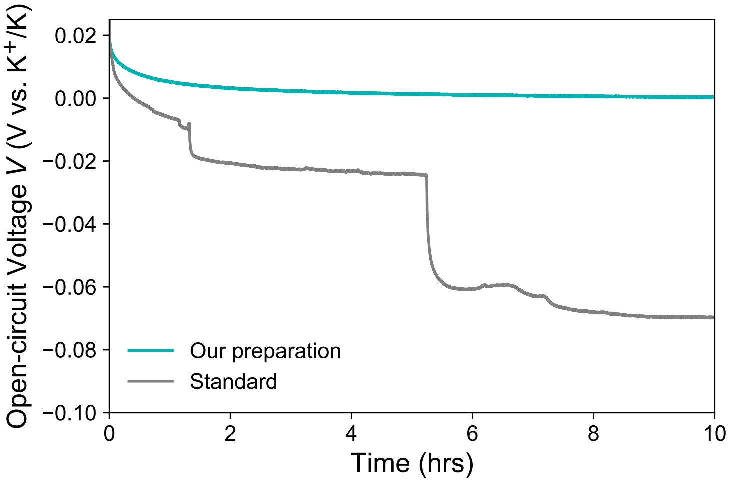

Supplementary Fig. 2 Diffusion open-circuit voltage relaxation profile after 10 h rest and 20 h galvanostatic polarisation in representative K||K symmetric cells in 1 m KFSI:DME in our restricted diffusion cell (Supplementary Fig. 19) using our K preparation compared to standard K preparation (Methods) at 20°C

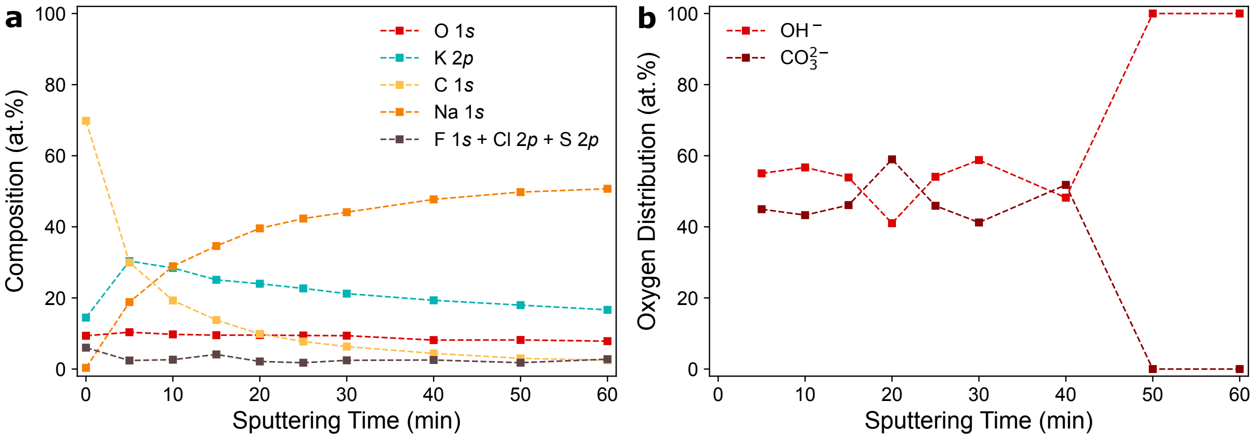

Supplementary Fig. 3 Quantification of the composition of the K metal surface as a function of sputtering time during X-ray photoelectron spectroscopy depth profiling on a K metal electrode produced using the standard preparation, where as-received K metal chunks are rinsed with hexane, rolled, and punched (Methods) (**a**) overall composition (**b**) breakdown of oxygen species

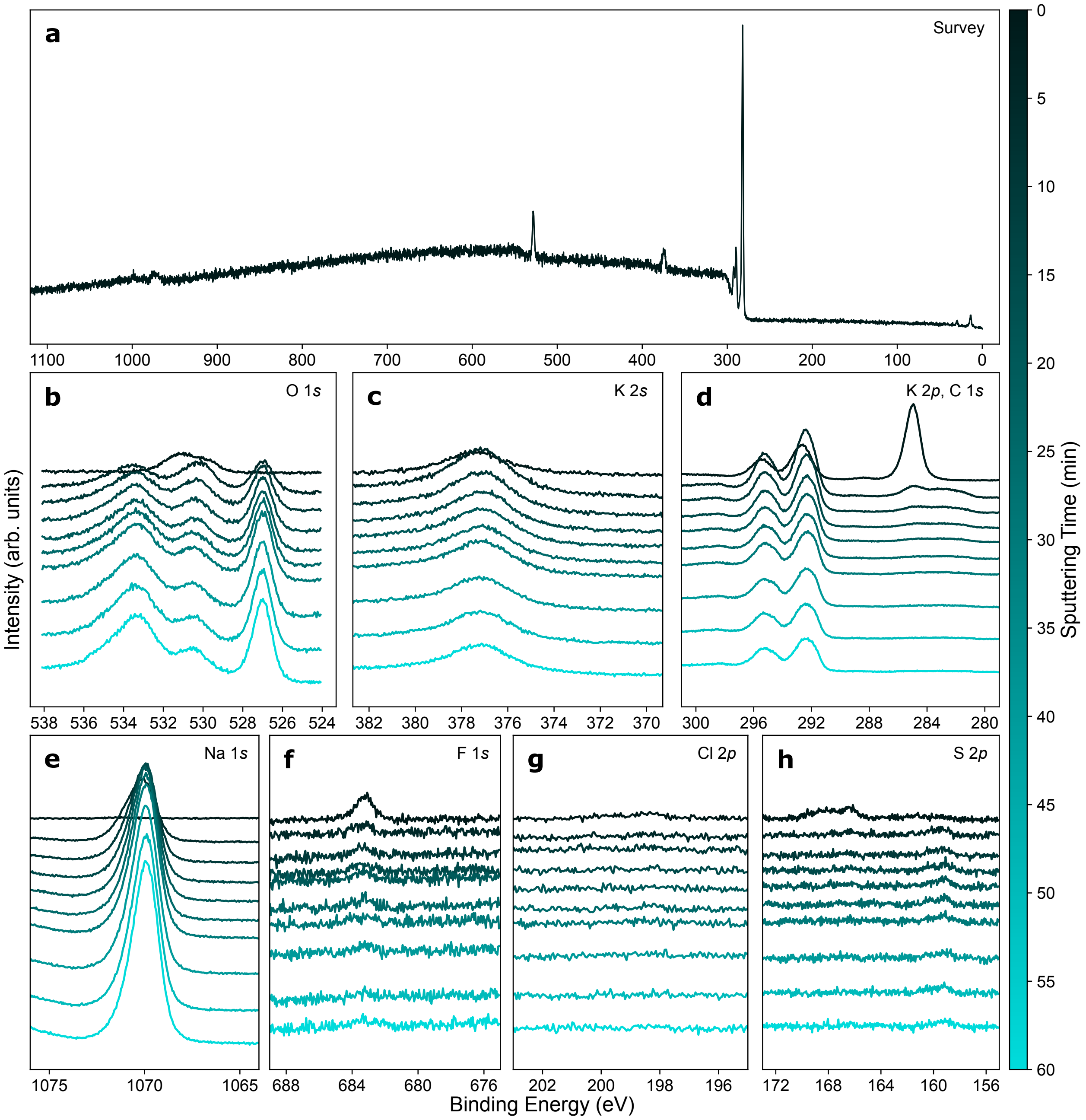

Supplementary Fig. 4 X-ray photoelectron spectroscopy depth profiles from a K metal electrode produced using the standard preparation, where as-received K metal chunks are rinsed with hexane, rolled, and punched (Methods) (**a**) survey (**b**) O 1*s* (**c**) K 2*s* (**d**) K 2*p*, C 1*s* (**e**) Na 1*s* (**f**) F 1*s* (**g**) Cl 2*p* (**h**) S 2*p*

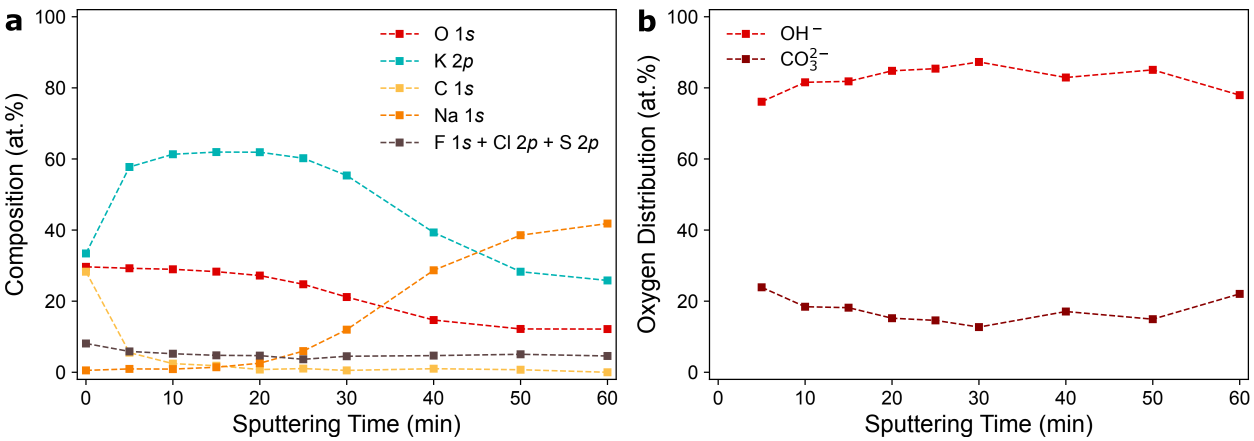

Supplementary Fig. 5 Quantification of the composition of the K metal surface as a function of sputtering time during X-ray photoelectron spectroscopy depth profiling on a K metal electrode produced using our K preparation method, where K is processed as described in Fig. 1 (Methods) (**a**) overall composition (**b**) breakdown of oxygen species

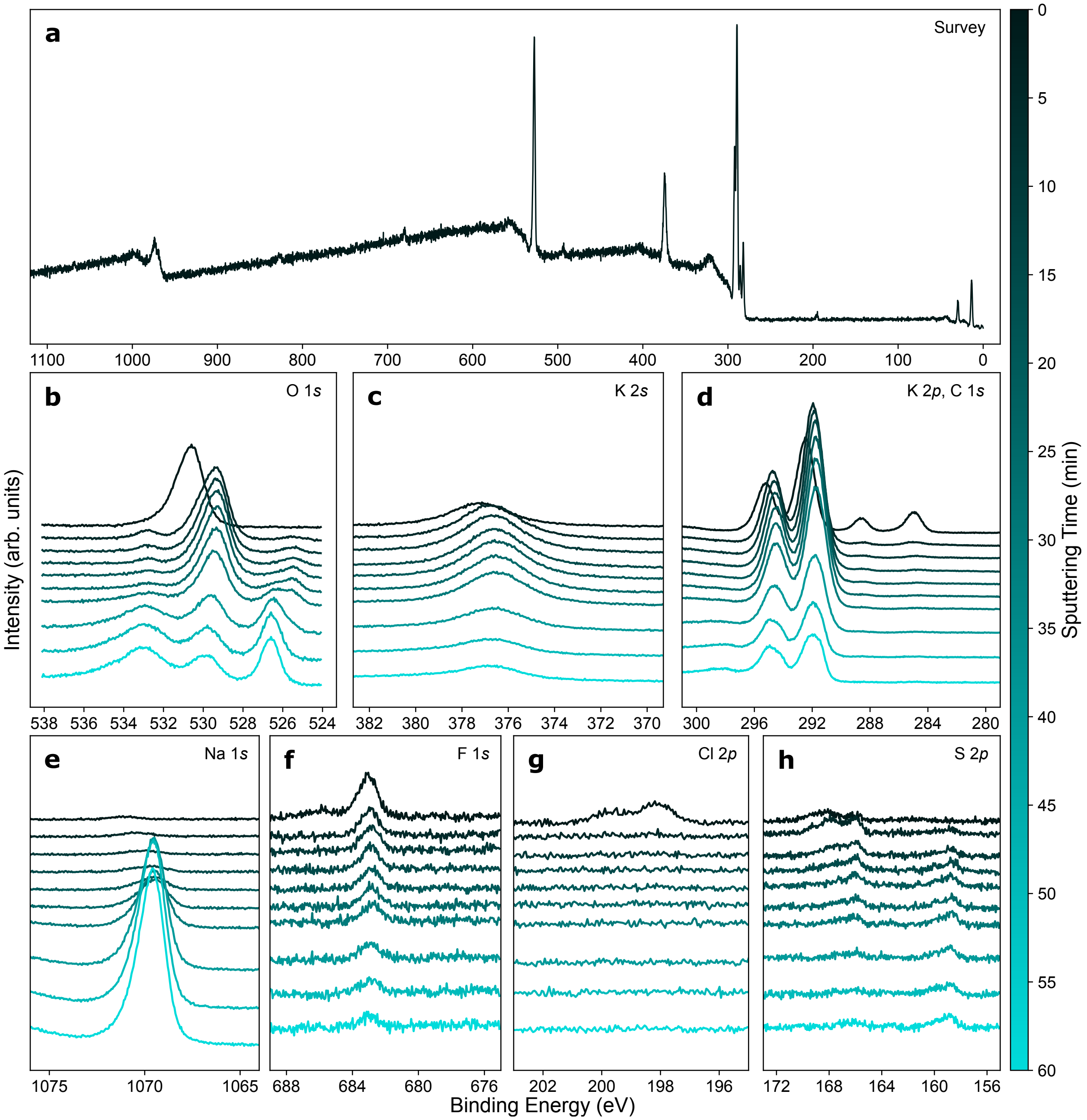

Supplementary Fig. 6 X-ray photoelectron spectroscopy depth profiles from a K metal electrode produced using our K preparation method, where K is processed as described in Fig. 1 (Methods) (**a**) survey (**b**) O 1*s* (**c**) K 2*s* (**d**) K 2*p*, C 1*s* (**e**) Na 1*s* (**f**) F 1*s* (**g**) Cl 2*p* (**h**) S 2*p*

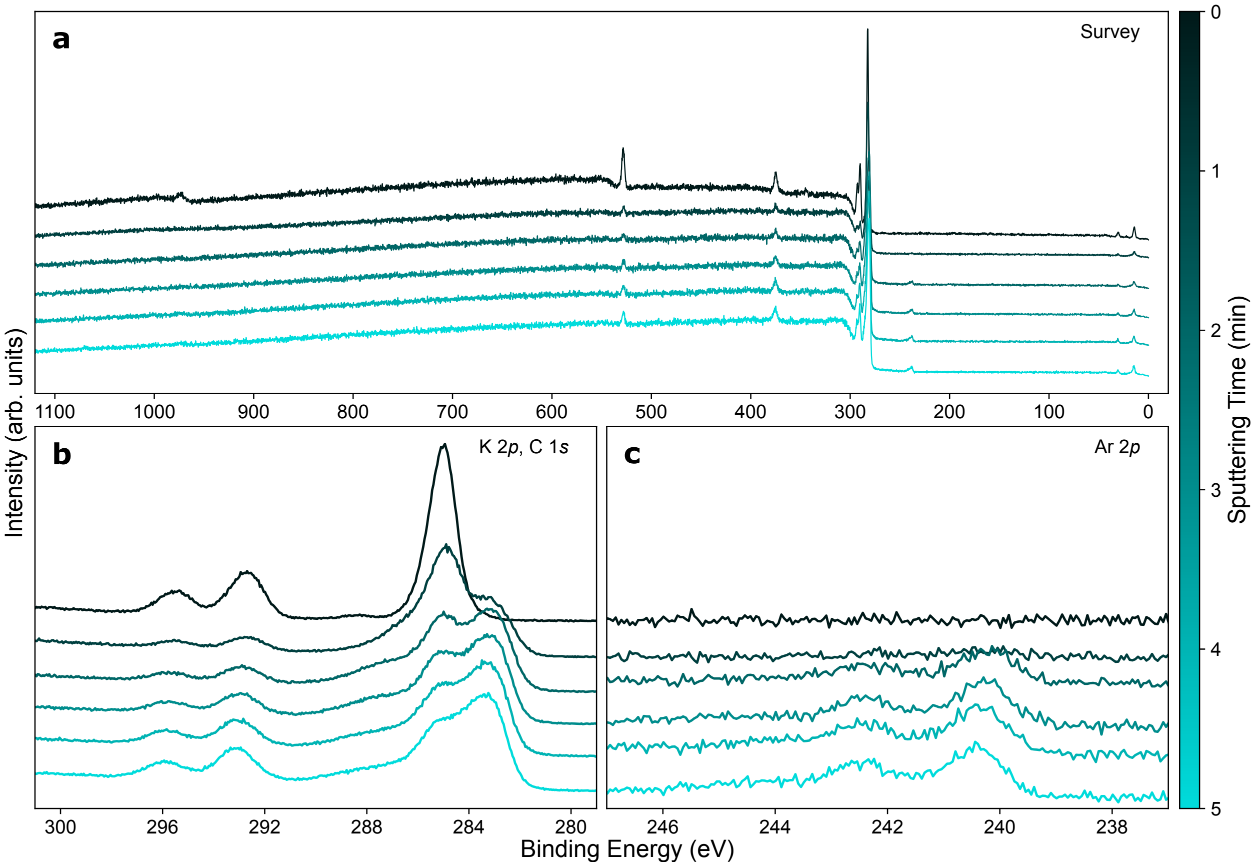

Supplementary Fig. 7 X-ray photoelectron spectroscopy depth profiles to determine the origin of the carbide species at the surface of a K metal electrode produced using the standard preparation, where as-received K metal chunks are rinsed with hexane, rolled, and punched (Methods) (**a**) survey (**b**) K 2*p*, C 1*s* (**c**) Ar 2*p*

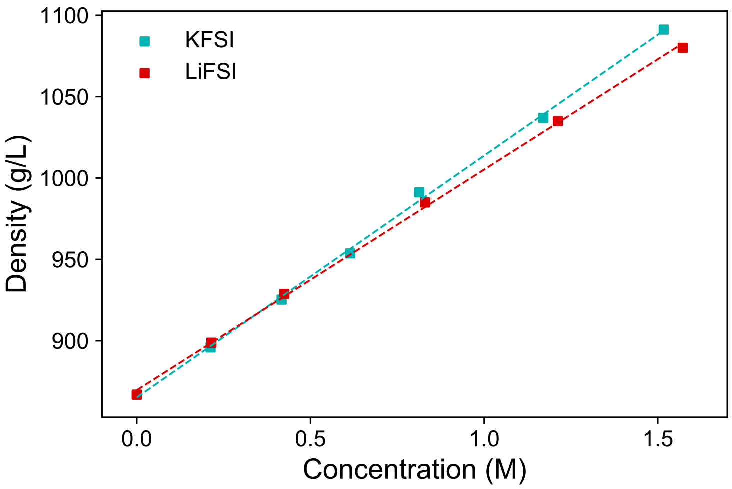

Supplementary Fig. 8 Density correlations for KFSI and LiFSI in DME at 20°C

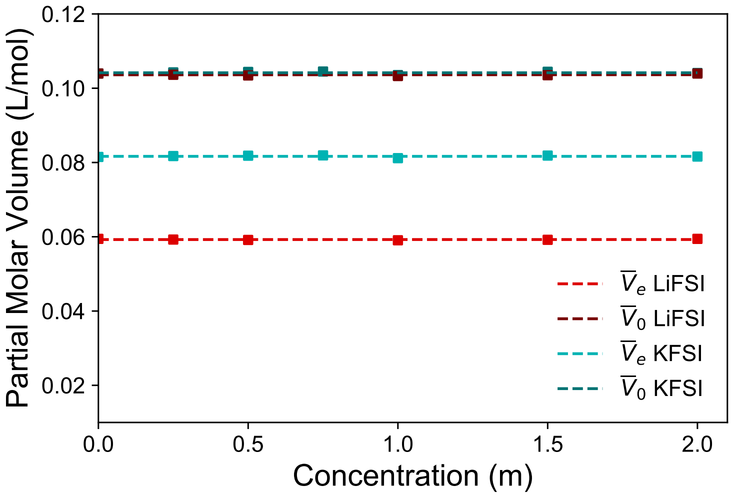

Supplementary Fig. 9 Partial molar volumes of salt, $\overline{V}_e$, and solvent, $\overline{V}_0$, for LiFSI and KFSI in DME at 20°C

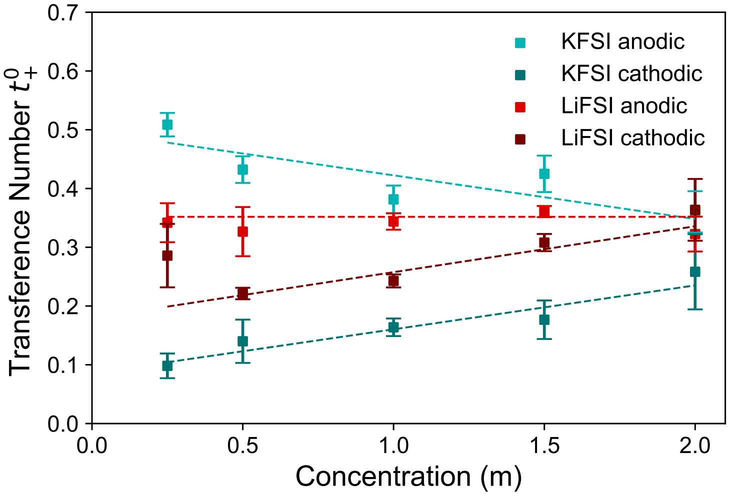

Supplementary Fig. 10 LiFSI and KFSI cation transference numbers determined from the Hittorf anodic and cathodic chambers in DME at 20°C. Error bars depict error in the mean (Supplementary Note 1)

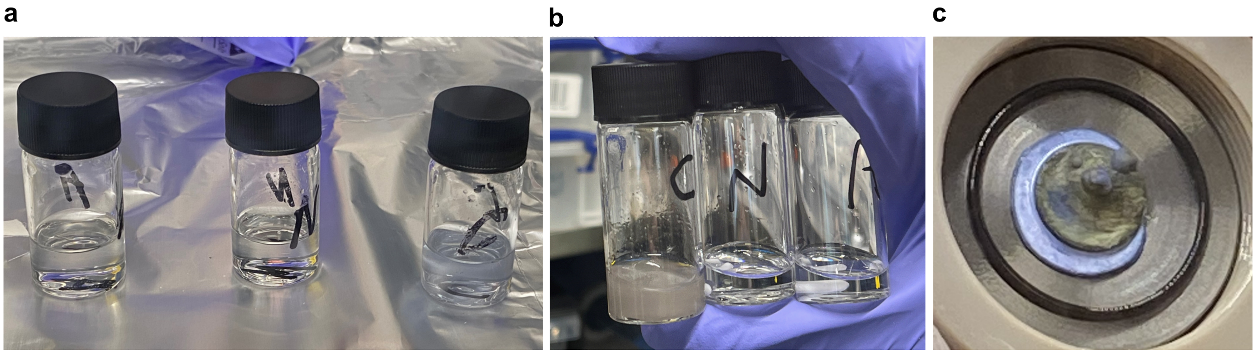

Supplementary Fig. 11 Cathodic chamber discolouration after Hittorf experiment in DME at 20°C. The cathodic, neutral and anodic solutions are labelled as C, N and A, respectively (**a**) KFSI cathodic solution with neutral and anodic solutions for comparison (**b**) LiFSI cathodic solution with neutral and anodic solutions for comparison (**c**) Cathodic Hittorf K electrode after 1 m KFSI:DME Hittorf experiment showing nonuniform plating

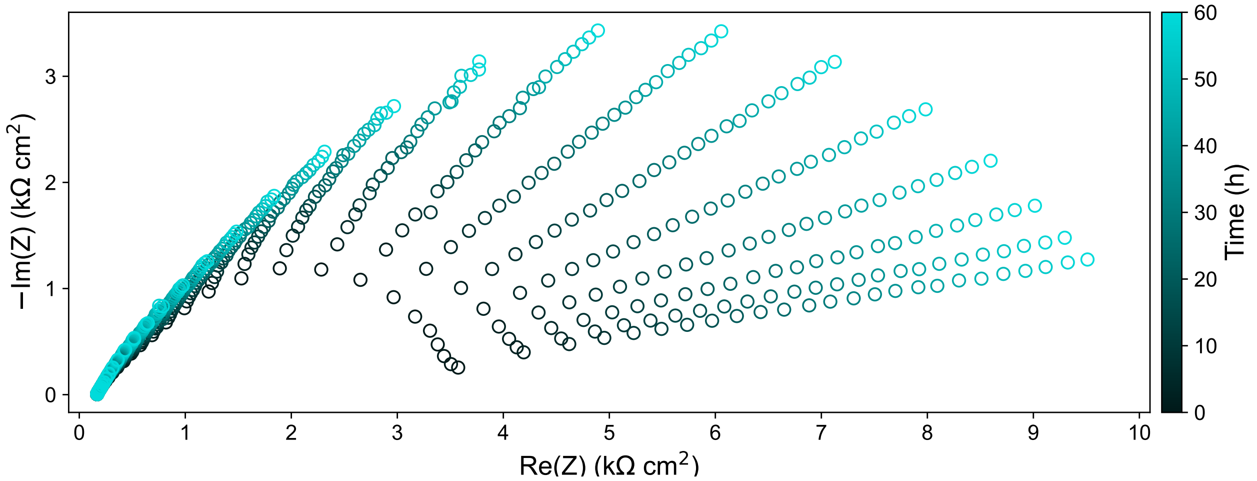

Supplementary Fig. 12 Complex impedance plot of K metal symmetric cell with 0.5 m KFSI in DME electrolyte over time during concentration gradient relaxation after a 20 h current pulse at 20°C

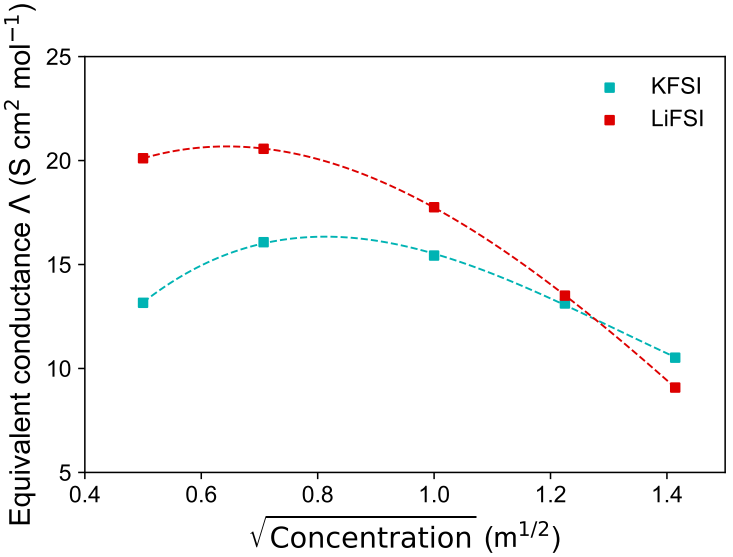

Supplementary Fig. 13 Equivalent conductance of KFSI and LiFSI in DME at 20°C

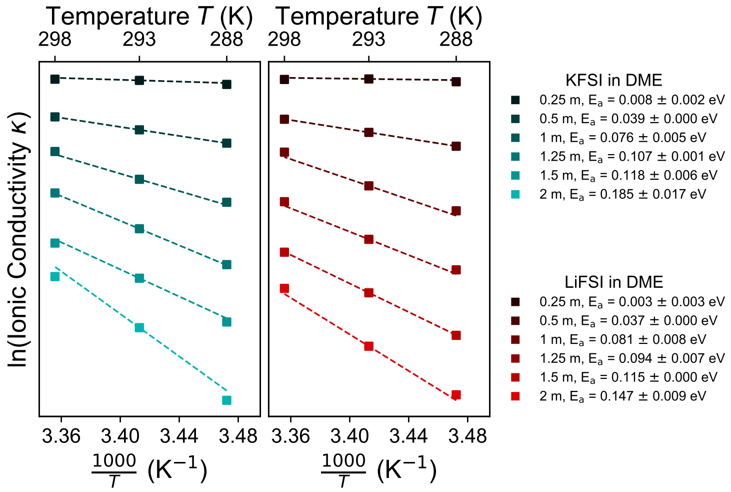

Supplementary Fig. 14 Arrhenius plots used to calculate the activation energy as a function of concentration. The different concentrations are shifted along the y-axis for clarity

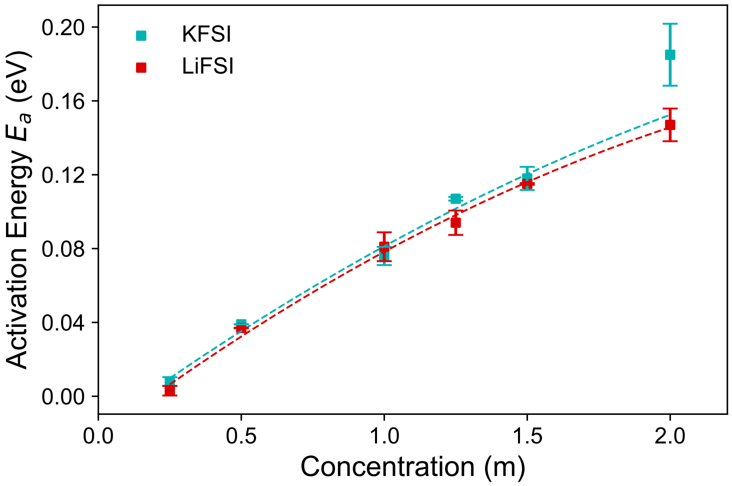

Supplementary Fig. 15 Activation energies for ionic conduction for KFSI and LiFSI in DME. Error bars depict the standard error in each activation energy determined from the linear fits in Supplementary Fig. 14

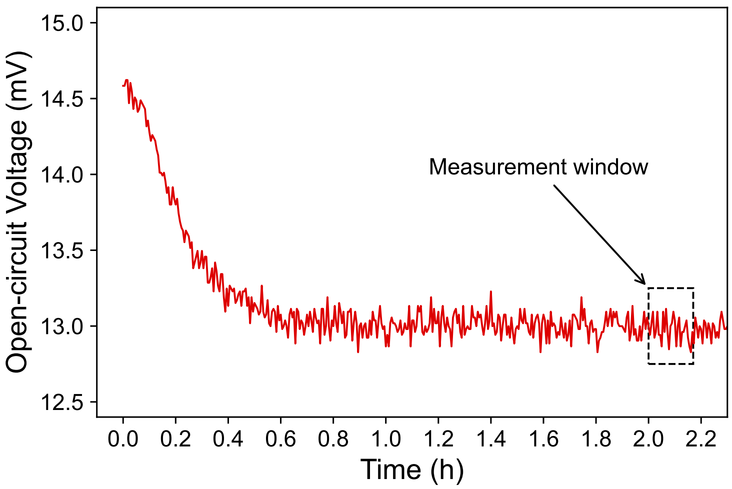

Supplementary Fig. 16 Open-circuit voltage (OCV) as a function of time for a representative concentration cell with a 1 m LiFSI in DME 'reference' concentration and a 1.5 m LiFSI in DME 'test' concentration at 20°C demonstrating the stabilisation of the OCV. The measurement window is visualised by the dashed box

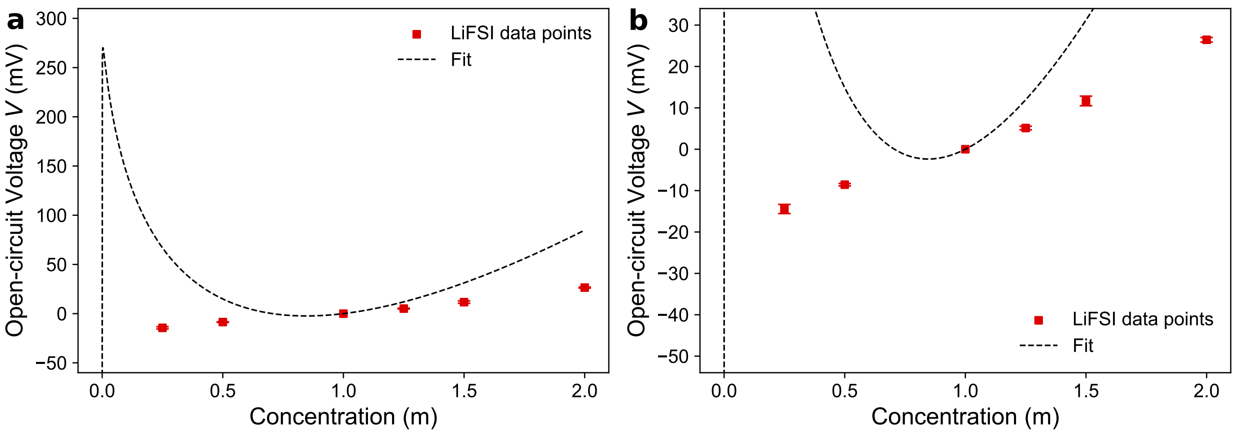

Supplementary Fig. 17 The fit to the LiFSI concentration cell open-circuit voltage data measured at 20°C determined by Supplementary Eq. 13 (**a**) full scale (**b**) reduced scale. Error bars depict the standard error in the mean (Supplementary Note 1)

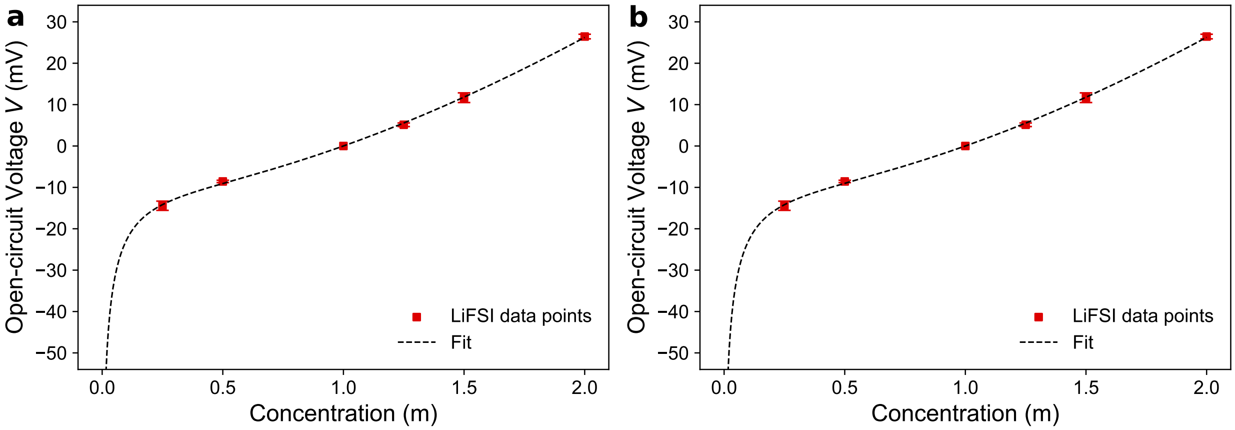

Supplementary Fig. 18 The fit to the LiFSI concentration cell open-circuit voltage data measured at 20°C determined by (**a**) Supplementary Eq. 14 (**b**) Supplementary Eq. 15. Error bars depict the standard error in the mean (Supplementary Note 1)

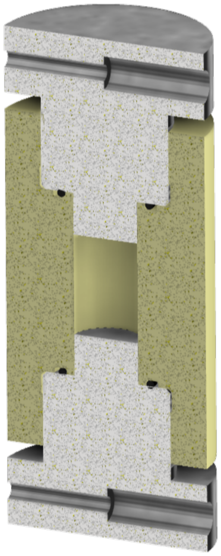

Supplementary Fig. 19 The designed restricted diffusion cell with polyether ether ketone (PEEK) chamber, stainless steel current collectors and perfluoroelastomer (FFKM) chemically resistant O-rings

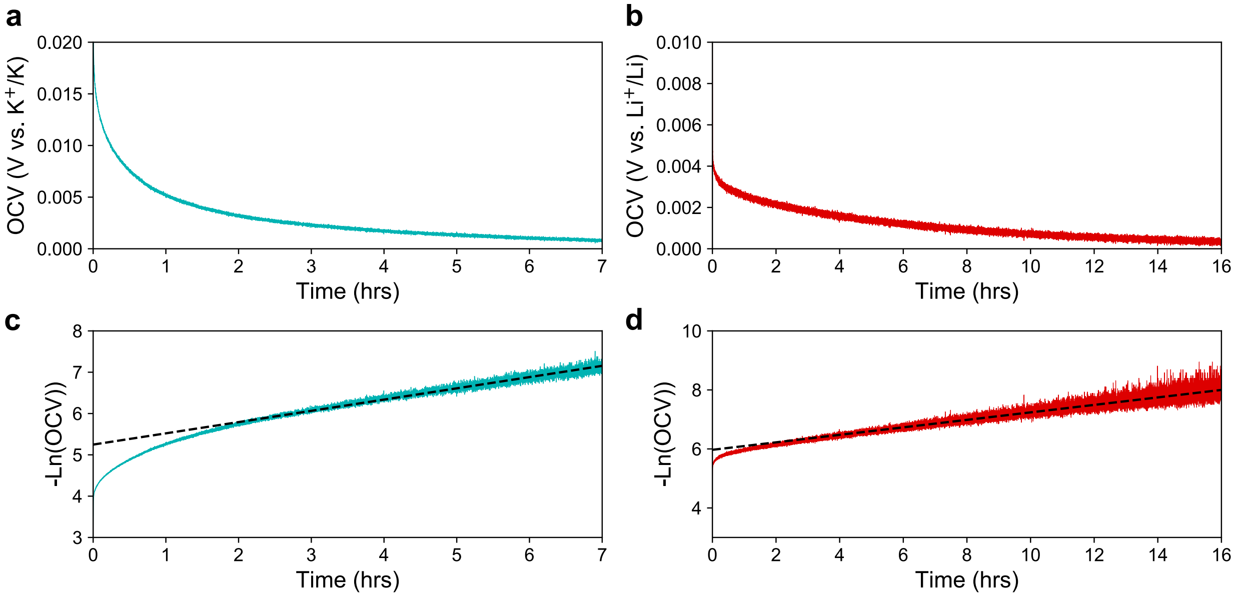

Supplementary Fig. 20 Representative restricted diffusion open-circuit voltage relaxation data for 1 m in DME at 20°C (**a**) KFSI (**b**) LiFSI. Open-circuit voltage data adjusted for V_{offset}. Semi-log plot with fitted linear decay region (black dashed line) used to determine salt diffusion coefficient (**c**) KFSI (**d**) LiFSI

.

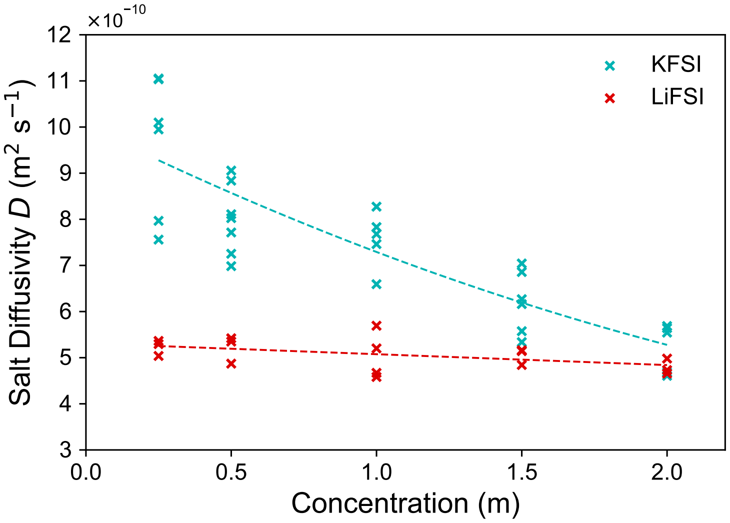

Supplementary Fig. 21 All salt diffusion coefficient results for KFSI and LiFSI in DME at 20°C

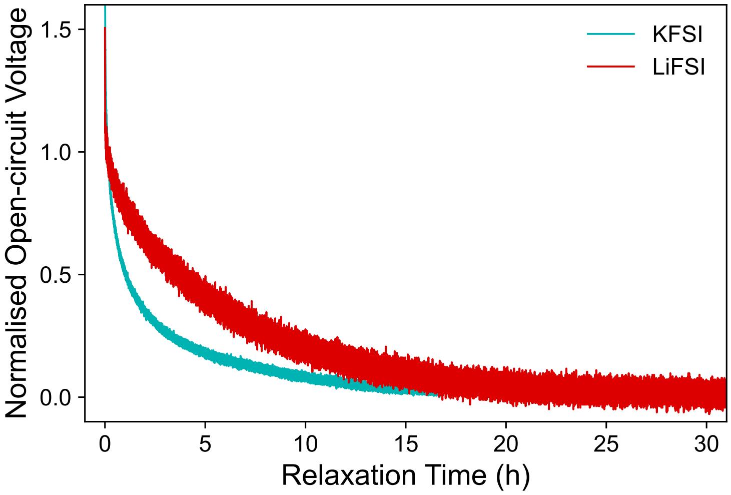

Supplementary Fig. 22 Relaxation profiles of 0.25 m LiFSI and KFSI in DME at 20°C, normalised to the potential after 100 s to exclude double layer effects, demonstrating faster relaxation in KFSI

Supplementary Note 1: Error Analysis

The error bars in Fig. 2a, 4a and 5a represent the standard error in the mean, $\sigma_{\overline{x}}$, estimated from the sample standard deviation, σ_x, and the number of data points, n, using Supplementary Eq. 1:

$$\sigma_{\overline{x}} = \frac{\sigma_x}{\sqrt{n}} \tag{1}$$

Due to the high sensitivity of the calculated transference numbers to changes in solution densities, the limited resolution of the densitometer results in transference numbers with a non-negligible uncertainty. Each calculated transference number can take any value between $t^0_{+,min}$ and $t^0_{+,max}$, with equal probability, giving each measurement, i, an uncertainty variance, σ_i^2, of $\frac{1}{12}(t^0_{+,max} - t^0_{+,min})^2$. The error in the mean t^0_+, $\sigma_{\overline{x}}$, was then estimated by taking into account the sample standard deviation, σ_x, and the uncertainty variance, σ_i^2, for each of the n data points through Supplementary Eq. 2:

$$\sigma_{\overline{x}} = \sqrt{\frac{\sigma_x^2}{n} + \frac{1}{n^2}\sum_{i=1}^{n}\sigma_i^2} \tag{2}$$

Estimated errors in D and t_+^0 were then propagated through to χ_M, and the thermodynamic and Stefan-Maxwell diffusivities using the standard propagation rules given in Supplementary Eq. 3:

$$x = \frac{uv}{w}, \quad \sigma_x = x\sqrt{\left(\frac{\sigma_u}{u}\right)^2 + \left(\frac{\sigma_v}{v}\right)^2 + \left(\frac{\sigma_w}{w}\right)^2} \tag{3}$$

Supplementary Note 2: Potassium Metal Electrodes

Characterising the electrolyte D using the leading restricted diffusion method requires exponential OCV relaxation after polarisation of symmetric metal cells (Supplementary Note 8). Supplementary Fig. 2 shows our K preparation results in exponential OCV relaxation in K symmetric cells, enabling the determination of D from its semi-log plot (Supplementary Fig. 20). The standard preparation cannot be used to determine D due to random OCV jumps, irreproducibility and erratic OCV behaviour, indicating side reactions and K instability, matching similar findings occurring for Na-ion electrolytes.[1]

Supplementary Note 3: X-ray Photoelectron Spectroscopy

The O 1s spectra from the standard preparation in Fig. 2c exhibits peaks at 533.6 and 527.0 eV as a result of Na KLL Auger electron emission and their relatively large areas are consistent with the large Na 1s peak evident in Supplementary Fig. 4 that increases with sputtering time, and Na is the main species identified throughout the majority of the examined depth (Supplementary Fig. 3a). The two O 1s peaks at binding energies of 529.9 and 530.7 eV are attributed to hydroxide and carbonate environments, respectively. The binding energy of the hydroxide peak is consistent with NaOH, while the carbonate peak

appears at a binding energy between those of the K_2CO_3 and Na_2CO_3 compounds, suggesting both may be present but cannot be resolved.[2]

The C 1s XPS spectra in Fig. 2c demonstrate a very minor carbonate peak around 289 eV and an adventitious C peak at 285 eV. There is an additional broad C 1s peak at 282.7 eV, which is characteristic of a carbide species. We performed additional XPS experiments to uncover the origin of this peak. Supplementary Fig. 7 presents the XPS spectra obtained during Ar^+ depth profiling for a total of 5 minutes. It is evident from the C 1s spectra in Supplementary Fig. 7b that this peak once again emerges, appearing as a shoulder on the adventitious carbon peak after 1 min of Ar^+ sputtering, and then increasing in intensity with increasing sputtering time. The only other peak that appears in the survey spectra during this period (Supplementary Fig. 7a) is the small peak at a binding energy of approximately 240 eV. Core-level spectra around this energy (Supplementary Fig. 7c) reveal the emergence of a doublet peak that is consistent with Ar 2p,[3] indicating that Ar implantation is taking place at the surface.

We have not detected any peaks corresponding to transition metals, so we conclude that a transition metal carbide cannot be the responsible species. We therefore suspect that the C 1s peak is instead due to either a K or Na acetylide species. Although there are few reports on these materials, the presence of Li_2C_2 has been confirmed in battery grade Li metal[4] and it generates a C 1s peak at a binding energy similar to that observed here.[5] It has been speculated that Li_2C_2 is present as a bulk impurity from Li production,[4] or that it forms due to a reaction between metallic Li and organic matter.[6] It is probable that similar processes could occur for Na or K, producing a carbide species that is responsible for the observed peak. Alternatively, a K or Na carbide species could form during the high-energy Ar^+ sputtering process. Ar-ion bombardment-induced mixing has been reported to induce carbide formation during the sputtering of Si[7] and Ti.[8] In this case, surface C species could be mixed with Na or K compounds during argon sputtering, promoting the formation of a

Na or K acetylide species.

The O 1s XPS spectra from K metal prepared with our method in Fig. 2d present only minor Na KLL Auger peaks, consistent with the much lower proportion of Na evident at the surface in Supplementary Fig. 5 and 6. The O 1s carbonate and hydroxide peaks appear at 530.4 and 529.3 eV, respectively, consistent with K_2CO_3 and KOH.[2,9] The C 1s spectra in Fig. 2d also show a minor carbonate peak at 288.4 eV and the adventitious C peak at 285 eV, with no evidence of a carbide species.

Supplementary Note 4: Hittorf Method and Transference Number

The Hittorf method is a technique which measures the change in moles of cations, compared to anions, across the cell with the passage of a set amount of charge. The transference number is directly related to the ratio between this change in moles and charge passed. The densitometric Hittorf cell, designed by Hou and Monroe, was used to characterise the transference number.[10] The large cell is isolated into three chambers using stopcocks after polarisation and the density differences between these chambers are measured to calculate the concentration differences and hence determine the transference number using Eq. 1.[10] The conditions used were an optimum to form a detectable density difference with low error whilst preventing the concentration boundary moving out of the anodic and cathodic chambers. The vertical orientation and direction of polarisation ensures cation flux directly opposes the direction of gravity, preventing the effects of natural convection affecting the measurement due to density gradients.[11]

Due to the volatility of DME, the Hittorf was checked that evaporation did not have any impact over the experiment by running a test cell with no metal electrodes or current applied, filled with electrolyte and measuring the resultant density of the electrolyte before and in each chamber after. There was no change in density showing DME evaporation is not an issue.

Supplementary Note 5: Partial Molar Volumes

The partial molar volumes of the salt, $\overline{V}_e$, and solvent, $\overline{V}_0$, are calculated from the densitometry results (Supplementary Fig. 8) using the following expressions:

$$\overline{V}_e = \frac{M_e - \frac{d\rho}{dc}}{\rho - c\frac{d\rho}{dc}} \tag{4}$$

$$\overline{V}_0 = \frac{M_0}{\rho - c\frac{d\rho}{dc}} \tag{5}$$

where M_e is the molar mass of KFSI/LiFSI, M_0 is the molar mass of DME, ρ is the density, c is the molar concentration and $\frac{d\rho}{dc}$ is the slope of the density correlation (Supplementary Fig. 8).

Supplementary Note 6: Ionic Conductivity and Activation Energy

Casteel-Amis Equation

The ionic conductivities in Fig. 3b were fitted with the function proposed by Casteel and Amis (Supplementary Eq. 6):[12]

$$\kappa(c) = \kappa_{max}\left(\frac{c}{c_{max}}\right)^{x_1}\exp\left(x_2(c - c_{max})^2 - \frac{x_1}{c_{max}}(c - c_{max})\right) \tag{6}$$

Where κ_{max} is the maximum ionic conductivity, c_{max} is the concentration at maximum ionic conductivity, and x_1 and x_2 are fitting constants.

Equivalent Conductance

The equivalent conductance, Λ, of both electrolytes was calculated from the ionic conductivity, κ, and molar concentration, c, using Supplementary Eq. 7[13] and is plotted in Supplementary Fig. 13.

$$\Lambda = \frac{\kappa}{z_+ \nu_+ c} \tag{7}$$

Where z_+ is the cation charge number and ν_+ is the number of cations into which the salt molecule dissociates.

Activation Energy

The activation energy, E_a, was calculated as a function of concentration from the conductivity measurements at three temperatures using Supplementary Eq. 8:[14]

$$\kappa = \kappa_0 e^{-\frac{E_a}{kT}} \tag{8}$$

Where κ_0 is a constant and k is the Boltzmann constant. The Arrhenius plots used to calculate E_a are shown in Supplementary Fig. 14 and the activation energies are plotted in Supplementary Fig. 15.

Supplementary Note 7: Concentration Cell and Thermodynamic Factor

A concentration cell places metallic electrodes into two electrolytes with the same constituents but different compositions in chemical contact, enabling measurement of the OCV that arises between them as a result of the difference in chemical potential from the different salt concentrations.[15] The concentration cell contains two chambers separated by a porous frit which enables electrochemical contact but significantly reduces interdiffusion. One chamber is filled with a 'reference' solution of constant concentration, while the other chamber contains a 'test' solution with a concentration that varies between experiments.

The thermodynamic factor, χ_M, is related to the change in the OCV measured across the concentration cells, V, with the molar concentration of the 'test' electrolyte, c, by

Supplementary Eq. 9:[15]

$$\chi_M = 1 + \frac{d\ln(f_\pm)}{d\ln(c)} = \frac{F}{2RT(1-t_+^0)}\frac{dV}{d\ln(c)} \tag{9}$$

Where $f_\pm$ is the mean molar activity coefficient, t_+^0 is the cation transference number, F is the Faraday constant, R is the gas constant and T is the absolute temperature. The solvent concentration, c_0, and the partial molar volume of the solvent, $\overline{V}_0$, can be used to map the thermodynamic factor to the molar basis from the molal basis in which it is defined.[16] It is also convenient to convert from molar concentration to molal concentration, c_m, resulting in Supplementary Eq. 10:

$$\chi_M = \frac{1}{c_0\overline{V}_0}\left(1 + \frac{d\ln(\gamma_\pm)}{d\ln(c_m)}\right) = \frac{F\frac{d\ln(c_m)}{d\ln(c)}}{2RT(1-t_+^0)}\frac{dV}{d\ln(c_m)} \tag{10}$$

Where $\gamma_\pm$ is the mean molal activity coefficient. For a strong, binary electrolyte $\gamma_\pm$ is predicted to vary with c_m according to Supplementary Eq. 11:[16]

$$\frac{d\ln(\gamma_\pm)}{d\ln(c_m)} = \frac{z_+z_-\alpha\sqrt{c_m}}{2(1+Ba\sqrt{c_m})^2} + A_2c_m + \frac{3A_3}{2}{c_m}^{\frac{3}{2}} + \cdots \tag{11}$$

Where the first term on the right-hand side comes from Debye-Hückel theory for the long-range electrical interactions between ions and the following terms account for additional concentrated-solution effects.[11] In Supplementary Eq. 11, z_i is the charge number of species i, α and B are constants given by Supplementary Eq. 12, a is the average ionic radius, and A_n are fitting constants.

$$\alpha = \frac{F^2e\sqrt{2\rho_0}}{8\pi(\varepsilon_r\varepsilon_0RT)^{3/2}}, \quad B = \frac{F\sqrt{2\rho_0}}{\sqrt{\varepsilon_r\varepsilon_0RT}} \tag{12}$$

In Supplementary Eq. 12 e is electronic charge, ρ_0 is solvent density, ε_r is relative permittivity of the solvent, and ε_0 is vacuum permittivity. Taking the values for DME ($\rho_0 =$

866.9 g L^{-1}, $\varepsilon_r = 7.2$) gives $\alpha = 40.38$ kg$^{\frac{1}{2}}$ mol$^{-\frac{1}{2}}$ and $B = 10.20$ kg$^{\frac{1}{2}}$ mol$^{-\frac{1}{2}}$ nm^{-1} at 20°C.

Combining Supplementary Eq. 10 and 11, and dropping terms beyond $A_2 c_m$[11] gives Supplementary Eq. 13:

$$\frac{dV}{d\ln(c_m)} = \frac{2RT(1-t_+^0)}{F\frac{d\ln(c_m)}{d\ln(c)}} \frac{1}{c_0\overline{V}_0}\left(1 + \frac{z_+ z_- \alpha\sqrt{c_m}}{2(1+Ba\sqrt{c_m})^2} + A_2 c_m\right) \tag{13}$$

In both electrolyte systems investigated $\frac{d\ln(c_m)}{d\ln(c)}$ was found to be constant and $\frac{1}{c_0\overline{V}_0}$ was well described by a function of the form $\frac{1}{c_0\overline{V}_0} = 1 + B_1 c_m$, where B_1 is a constant. Supplementary Eq. 13 was integrated with respect to $\ln(c_m)$, with the boundary condition that $V = 0$ V at $c_m = 1$ m, to find an expression for V as a function of c_m that was then fit to the OCV data.

This function is fit to the LiFSI data in Supplementary Fig. 17, taking t_+^0 to be constant (Fig. 3a) and assuming $a = 0.76$ Å, the ionic radius of Li^+.[17] However, this resulted in a poor fit to the data.

Debye-Hückel theory assumes electrolytes are strong and fully dissociate,[11,13] which we have demonstrated not to be the case for the electrolytes studied here. This can be taken into account by introducing a second fitting constant, ξ,[13] to Supplementary Eq. 13, resulting in Supplementary Eq. 14.

$$\frac{dV}{d\ln(c_m)} = \frac{2RT(1-t_+^0)}{F\frac{d\ln(c_m)}{d\ln(c)}} \frac{1}{c_0\overline{V}_0}\left(1 + \frac{z_+ z_- \alpha\sqrt{\xi c_m}}{2(1+Ba\sqrt{\xi c_m})^2} + A_2 c_m\right) \tag{14}$$

ξ can be thought of as a correction factor to the ionic strength, and its introduction leads to a much better fit to the data, as evident in Supplementary Fig. 18a.

Additionally, the value of ξ determined by the fit is such that $1 + Ba\sqrt{\xi c_m} \approx 1$, allowing Supplementary Eq. 14 to be simplified to Supplementary Eq. 15:

$$\frac{dV}{d\ln{(c_m)}} = \frac{2RT}{F\frac{d\ln{(c_m)}}{d\ln{(c)}}}(1-t_+^0)\frac{1}{c_0\overline{V}_0}\left(1+A_1{c_m}^{\frac{1}{2}}+A_2c_m\right) \quad (15)$$

Where A_1 ($\approx \frac{z_+z_-\alpha\sqrt{\xi}}{2}$) is a fitting constant. Supplementary Eq. 15 results in just as good a fit to the data in Supplementary Fig. 18b, and allows the concentration dependence of $t^0_{\mathrm{K}^+}$ to be taken into account. Once A_1 and A_2 are fit to the OCV data, χ_M can be calculated from Supplementary Eq. 16:

$$\chi_M = \frac{1}{c_0\overline{V}_0}(1+A_1{c_m}^{\frac{1}{2}}+A_2c_m) \quad (16)$$

Supplementary Note 8: Restricted Diffusion and Salt Diffusivity

The restricted diffusion experiment[10,18–21] is the most accurate and reproducible method to measure electrolyte salt diffusion coefficient, D, involving a sealed vertically oriented cell where current/potential is applied so stripping occurs at the bottom. The vertical orientation and direction of polarisation again ensures cation flux directly opposes the direction of gravity, preventing the effects of natural convection affecting the measurement due to density gradients.[11] A concentration gradient is induced by galvanostatic polarisation, and the relaxation of the concentration gradient is measured between two points by tracking the OCV. D is determined from this exponential relaxation of OCV using Eq. 4.

In D analysis using the restricted diffusion method, there is some dependence of the resulting diffusion coefficient on the time window it is fit.[22] Newman and Thompson demonstrated that exponential decay after polarisation occurs for times greater than $0.05l^2/D$ ($\approx$ 0.5 τ_{diff}), regardless of the initial concentration profile indicating this is the point from which the data should be fit.[23] Therefore, in this analysis the data was fit from this minimum time constant 0.5 τ_{diff} for as long as it showed exponential relaxation behaviour, or until relaxation had completed. Due to the significantly higher diffusivity of KFSI than

LiFSI, this was on average 11 h for KFSI and 33 h for LiFSI. For K-ion electrolytes, due to the reactivity of the K metal, very long analysis time windows occasionally resulted in significant OCV data noise levels or random jumps in potential, similar to what was found for the Na-ion electrolyte $NaPF_6$:EC:DEC.[1] This is attributed to the continued reformation of the SEI on the freshly plated K metal affecting local ion concentration and increasing side reactions, as evidenced by the increasing impedance in Supplementary Fig. 12.

Supplementary Fig. 12 demonstrates the instability of the solid electrolyte interphase (SEI) that forms on K metal in KFSI:DME electrolytes. The impedance continuously increases over time, indicative of a growing SEI.[24] This suggests electrolyte reduction reactions continue to occur at the K metal-electrolyte interfaces. We found that the SEI was least stable at lower concentration, resulting in a higher impedance.

Thermodynamic Diffusivity

The thermodynamic diffusivity, $\mathscr{D}$, is calculated from D, χ_M, total molarity, c_T, and partial molar volume of solvent, $\overline{V}_0$, according to Supplementary Eq. 17:[15]

$$\mathscr{D} = \frac{D}{c_T \overline{V}_0 \chi_M} \tag{17}$$

Stefan-Maxwell Diffusivities

The Stefan-Maxwell diffusion coefficients provide a more detailed understanding of the diffusional behaviour of each individual electrolyte species and their interactions with other species in solution. The Stefan-Maxwell formalism expresses transport laws through the thermodynamic forces which drive diffusion.[10,13] The coefficients are calculated from the characterised transport properties using the following expressions:[11]

$$\mathscr{D}_{0-} = \frac{z_+}{z_+ - z_-} \frac{\mathscr{D}}{t_+^0} \tag{18}$$

$$\mathscr{D}_{0+} = \frac{-z_-}{z_+ - z_-} \frac{\mathscr{D}}{1 - t_+^0} \tag{19}$$

$$\frac{1}{\mathscr{D}_{+-}} = -\frac{z_+ z_- c_T F^2}{RT\kappa} - \frac{z_+ - z_-}{z_+ \nu_+} \frac{c_0 t_+^0 (1 - t_+^0)}{c\mathscr{D}} \tag{20}$$

Where $\mathscr{D}_{0-}$ and $\mathscr{D}_{0+}$ are the Stefan-Maxwell diffusion coefficients between the solvent and the anion and cation, respectively. $\mathscr{D}_{+-}$ is the Stefan-Maxwell diffusion coefficient between the cation and anion. c_T is the total particle molarity, $c_T = c_0 + c_- + c_+$, where c_0 is the solvent molarity ($c_0 = \frac{1-\overline{V}_e}{\overline{V}_0}$), and c_- and c_+ are the cation and anion molarity, respectively. z_i and ν_i are the charge number and the number of ions of species i into which the salt molecule dissociates, respectively.

Supplementary Note 9: Property Parameterisation

All transport and thermodynamic properties were parameterised by fitting functions to the data points. If the functional form was known (Supplementary Eq. 6 for κ) then non-linear least squares fitting was used. Otherwise, least squares polynomial fits were used, with inverse variance weighting for the case of t_+^0.

The parameterised forms of t_+^0, κ, χ_M and D are given as a function of concentration (either molar, c, or molal, c_m) for LiFSI and KFSI in DME, respectively. All fits are from data gathered in the concentration range 0.25–2 m.

Approximate conversions between molal and molar concentrations are also provided.

LiFSI in DME

Cation transference number, t_+^0:

$$t_+^0 = 0.352 \tag{21}$$

Ionic conductivity, κ (mS cm^{-1}):

$$\kappa = 16.286\left(\frac{c}{1.158}\right)^{1.085}\exp\left(-0.426(c-1.158)^2 - \frac{1.085}{1.158}(c-1.158)\right) \tag{22}$$

Thermodynamic factor, χ_M:

$$\chi_M = (1+0.052c_m)(1-2.663{c_m}^{\frac{1}{2}}+2.287c_m) \tag{23}$$

Salt diffusivity, D (m^2 s^{-1}):

$$D = 5.312\times10^{-10} - (2.358\times10^{-11})c_m \tag{24}$$

Concentration conversion:

$$c = 0.873c_m - 0.043{c_m}^2 \tag{25}$$

KFSI in DME

Cation transference number, t_+^0:

$$t_+^0 = 0.497 - 0.074c_m \tag{26}$$

Ionic conductivity, κ (mS cm^{-1}):

$$\kappa = 15.970\left(\frac{c}{1.493}\right)^{1.620}\exp\left(0.023(c-1.493)^2 - \frac{1.620}{1.493}(c-1.493)\right) \tag{27}$$

Thermodynamic factor, χ_M:

$$\chi_M = (1+0.071c_m)(1-1.833{c_m}^{\frac{1}{2}}+1.608c_m) \tag{28}$$

Salt diffusivity, D (m^2 s^{-1}):

$$D = 1.003 \times 10^{-9} - (3.104 \times 10^{-10})c_m + (3.633 \times 10^{-11}){c_m}^2 \tag{29}$$

Concentration conversion:

$$c = 0.859c_m - 0.051{c_m}^2 \tag{30}$$

Supplementary References

1. Landesfeind, J. *et al.* Comparison of Ionic Transport Properties of Non-Aqueous Lithium and Sodium Hexafluorophosphate Electrolytes. *J. Electrochem. Soc.* **168,** 040538 (2021).
2. Caracciolo, L., Madec, L. & Martinez, H. XPS Analysis of K-based Reference Compounds to Allow Reliable Studies of Solid Electrolyte Interphase in K-ion Batteries. *ACS Appl. Energy Mater.* **4,** 11693–11699 (2021).
3. Crist, B. V. Argon Implanted into Graphite, by XPS. *Surf. Sci. Spectra* **1,** 376–380 (1992).
4. Schmitz, R. *et al.* Investigation of Lithium Carbide Contamination in Battery Grade Lithium Metal. *J. Power Sources* **217,** 98–101 (2012).
5. Kanamura, K., Shiraishi, S., Tamura, H. & Takehara, Z. X-Ray Photoelectron Spectroscopic Analysis and Scanning Electron Microscopic Observation of the Lithium Surface Immersed in Nonaqueous Solvents. *J. Electrochem. Soc.* **141,** 2379–2385 (1994).
6. Fonseca Rodrigues, M.-T. *et al.* Lithium Acetylide: A Spectroscopic Marker for Lithium Deposition During Fast Charging of Li-Ion Cells. *ACS Appl. Energy Mater.* **2,** 873–881 (2018).
7. Pan, J. S., Wee, A. T. S., Huan, C. H. A., Tan, H. S. & Tan, K. L. Argon Incorporation and Silicon Carbide Formation During Low Energy Argon-Ion Bombardment of Si(100). *J. Appl. Phys.* **79,** 2934–2941 (1996).
8. Luthin, J., Plank, H., Roth, J. & Linsmeier, C. Ion Beam-Induced Carbide Formation at the Titanium–Carbon Interface. *Nucl. Instrum. Methods Phys. Res. B* **182,** 218–226 (2001).
9. Allgayer, F., Maibach, J. & Jeschull, F. Comparing the Solid Electrolyte Interphases on Graphite Electrodes in K and Li Half Cells. *ACS Appl. Energy Mater.* **5,** 1136–1148 (2022).

10. Hou, T. & Monroe, C. W. Composition-dependent Thermodynamic and Mass-transport Characterization of Lithium Hexafluorophosphate in Propylene Carbonate. *Electrochim. Acta* **332,** 135085 (2020).

11. Newman, J. & Thomas-Alyea, K. E. *Electrochemical Systems, 3rd Edition* (Wiley, 2004).

12. Casteel, J. F. & Amis, E. S. Specific Conductance of Concentrated Solutions of Magnesium Salts in Water-Ethanol System. *J. Chem. Eng. Data* **17,** 55–59 (1972).

13. Wang, A. A., Hou, T., Karanjavala, M. & Monroe, C. W. Shifting-reference Concentration Cells to Refine Composition-dependent Transport Characterization of Binary Lithium-ion Electrolytes. *Electrochim. Acta* **358,** 136688 (2020).

14. Yim, C.-H. & Abu-Lebdeh, Y. A. Connection between Phase Diagram, Structure and Ion Transport in Liquid, Aqueous Electrolyte Solutions of Lithium Chloride. *J. Electrochem. Soc.* **165,** A547–A556 (2018).

15. Wang, A. A. *et al.* Review of Parameterisation and a Novel Database (LiionDB) for Continuum Li-ion Battery Models. *Prog. Energy* **4,** 032004 (2022).

16. Stewart, S. & Newman, J. Measuring the Salt Activity Coefficient in Lithium-battery Electrolytes. *J. Electrochem. Soc.* **155,** A458 (2008).

17. Dhir, S., Wheeler, S., Capone, I. & Pasta, M. Outlook on K-Ion Batteries. *Chem* **6,** 2442–2460 (2020).

18. Harned, H. S. & French, D. M. A Conductance Method for the Determination of the Diffusion Coefficients of Electrolytes. *Ann. NY Acad. Sci.* **46,** 267–284 (1945).

19. Newman, J. & Chapman, T. W. Restricted Diffusion in Binary Solutions. *AIChE J* **19,** 343–348 (1973).

20. Ma, Y. *et al.* The Measurement of a Complete Set of Transport Properties for a Concentrated Solid Polymer Electrolyte Solution. *J. Electrochem. Soc.* **142,** 1859–1868 (1995).

21. Ehrl, A., Landesfeind, J., Wall, W. A. & Gasteiger, H. A. Determination of Transport Parameters in Liquid Binary Lithium Ion Battery Electrolytes. *J. Electrochem. Soc.* **164,** A826–A836 (2017).

22. Bergstrom, H. K., Fong, K. D. & McCloskey, B. D. Interfacial Effects on Transport Coefficient Measurements in Li-ion Battery Electrolytes. *J. Electrochem. Soc.* **168,** 060543 (2021).

23. Thompson, S. D. & Newman, J. Differential Diffusion Coefficients of Sodium Polysulfide Melts. *J. Electrochem. Soc.* **136,** 3362–3369 (1989).

24. Nojabaee, M., Kuster, K., Starke, U., Popovic, J. & Maier, J. Solid Electrolyte Interphase Evolution on Lithium Metal in Contact with Glyme-Based Electrolytes. *Small* **16,** e2000756 (2020).
